# Analysis of a Medically Certified, Wrist-Worn Sensor for the Assessment of Heart Rate and Energy Expenditure During Daily Activities in Patients With Chronic Heart Failure or Coronary Artery Disease and Recreational Athletes: Validation Study

**DOI:** 10.2196/69343

**Published:** 2025-09-30

**Authors:** Ignace L J De Lathauwer, Valerie A A van Es, Mayke M C J van Leunen, Steven Onkelinx, Rutger W M Brouwers, Danny A J P van de Sande, Mathias Funk, Hareld M C Kemps

**Affiliations:** 1 Department of Cardiology, Máxima Medisch Centrum, De Run 4600, Veldhoven, 5504DB, The Netherlands, +31 40 888 8000; 2 Department of Industrial Design, Eindhoven University of Technology, Eindhoven, The Netherlands; 3 MoMiLab research unit, IMT School for Advanced Studies Lucca, Lucca, Italy; 4 Department of Allied Health Professions, Fontys University of Applied Sciences, Eindhoven, The Netherlands

**Keywords:** cardiac diseases, noninvasive device, smartwatch, validation studies, monitoring cardiac patients

## Abstract

**Background:**

Exercise capacity and lifestyle have proven to be important prognostic factors for cardiovascular patients. Both can be ameliorated through different preventive interventions. Cardiac rehabilitation and remote patient monitoring have been proven to reduce cardiac events and cardiovascular mortality. One of the most important goals of cardiac rehabilitation and remote patient monitoring is improving physical fitness and monitoring of cardiovascular parameters, which could predict cardiac deterioration. In order to monitor cardiac patients successfully, reliable and nonobtrusive devices to assess physical activity and cardiovascular parameters need to be available.

**Objective:**

This validation study aims to determine the accuracy of the Philips Health Band (PHB), a noninvasive, wrist-worn, medically certified device, for the assessment of heart rate (HR) and energy expenditure (EE) in patients with chronic cardiovascular diseases and recreational athletes (RAs).

**Methods:**

The assessment of HR and EE by the PHB was compared with indirect calorimetry (Oxycon Mobile [OM; CareFusion GmbH]) during an activity protocol consisting of daily activities. Three groups were assessed: patients with heart failure with reduced ejection fraction (HFrEF), patients with stable coronary artery disease (CAD) with preserved left ventricular ejection fraction, and RAs.

**Results:**

A total of 57 patients were included: 19 with CAD, 19 with HFrEF, and 19 RAs. HR assessment in the HFrEF and CAD groups was significantly underestimated over the entire protocol by the PHB as compared to the OM, with poor and fair reliability, respectively. No significant difference in HR was found between the PHB and OM over the entire protocol for the RA group, with good reliability (HFrEF: mean difference 3.0; *P*<.001; intraclass correlation coefficient [ICC] 0.36; CAD: mean difference 2.7; *P*<.001; ICC 0.55; RA: mean difference 0.8; ICC 0.60). Assessment of EE showed an underestimation over the entire protocol for the RA and CAD group, with poor and fair reliability, respectively. The HFrEF group showed no significant difference in EE assessment over the entire protocol, with poor reliability (HFrEF: mean difference 0.09; ICC 0.32; CAD: mean difference 0.29; *P*<.001; ICC 0.46; RA: mean difference 0.79; *P*<.001; ICC 0.26). The responsiveness to detect within-patient changes in activity intensity of the PHB was moderate for the HFrEF and CAD groups and acceptable for the RA group.

**Conclusions:**

HR and EE assessment of a medically certified noninvasive sensor using a photoplethysmogram and accelerometer showed poor accuracy and moderate responsiveness during an activity protocol reflecting daily living activities in patients with stable CAD and chronic HFrEF. Accuracy of HR in RAs was good and the responsiveness for both HR and EE was acceptable. This research confirms previous research and stresses the need for better patient-specific algorithms in noninvasive sensors, taking cardiovascular pathology and medication usage into account, for assessing HR and EE prior to their implemention in patient care.

## Introduction

Exercise capacity is known to be an important prognostic factor in patients with cardiovascular disease. Coronary artery disease (CAD) and heart failure (HF) are two of the most prevalent cardiovascular diseases, affecting millions of people worldwide [1]. In patients with CAD, regular moderate-intensity physical activity (PA) is associated with an increase in peak aerobic capacity and a reduction in all-cause mortality [2]. Research has shown a similar effect for patients with HF, indicating that decreased exercise capacity is linked to an increased risk of atrial arrhythmias, mortality, and hospitalizations due to HF exacerbations [3]. A study demonstrated that, even after adjusting for age, exercise capacity remains the strongest predictor for risk of death in both patients with cardiovascular disease and healthy individuals undergoing exercise testing. Due to this, exercise capacity is a more powerful predictor for mortality among men than other established risk factors of cardiovascular disease [4].

Over the years, several preventive interventions have emerged to enhance the prognosis of patients with cardiovascular disease by coaching as well as monitoring their health status [5]. First, exercise-based cardiac rehabilitation (CR) after an acute coronary syndrome is associated with a reduction of the risk of repeated cardiac events and cardiovascular mortality [6,7]. Despite the benefits of CR, participation rates remain low due to factors such as long distances to CR facilities and patient age [8]. Consequently, telerehabilitation has been proposed as an innovative solution. Second, research has demonstrated that remote patient monitoring (RPM) of patients with HF is effective in reducing mortality and HF-related hospitalizations [9-12]. Current RPM interventions use spot measurements of weight, blood pressure, and heart rate (HR) to monitor patients. In order to optimize RPM interventions and enable telerehabilitation, noninvasive sensors are needed for continuous monitoring of cardiovascular parameters.

It is essential for a monitoring device to be accurate and responsive if implemented in patient care. The accuracy of a device is defined as the closeness of agreement between the monitoring device measurement and the true value [13]. Responsiveness of a device is defined as its ability to detect within-patient changes of exercise intensity or cardiovascular parameters over time and is therefore highly important in patients with cardiovascular disease to monitor progression or their overall health status.

Previous trials investigating commercially available sensors in healthy individuals have shown mixed results and cannot be directly extrapolated to patients with cardiovascular disease due to differences in cardiac function and medication use, which affect chronotropic competence [14-17]. This emphasizes the need for validation studies in patients with cardiovascular disease. Herkert et al [18] demonstrated that 2 wrist-worn devices performed poorly in estimating energy expenditure (EE) and detecting within-patient changes during low-to-moderate exercise intensities in patients with HF and CAD. Similarly, a study evaluating the first generation Apple Watch, in patients with cardiovascular disease, found clinically acceptable HR accuracy during exercise, but an overestimation of EE [19]. A recent systematic review demonstrated that while Fitbit devices accurately measured step count and Apple Watch reliably measured HR, none of the tested devices accurately estimated EE, and most were not validated in patients with cardiovascular disease [20]. These findings suggest ongoing technical progress but emphasize the need for population-specific validation before such devices can be reliably used in patient care.

The aim of this validation trial is to investigate the accuracy and responsiveness of a medically certified wrist-worn sensor, the Philips Health Band (PHB), for the assessment of HR and EE in 3 patient populations: patients with HF with reduced ejection fraction (HFrEF), patients with stable CAD and preserved left ventricular ejection fraction (LVEF), and recreational athletes (RAs). If the PHB shows sufficient accuracy and responsiveness for measuring HR and EE, and thus PA levels, it could be implemented in clinical care (eg, telerehabilitation, secondary prevention, and RPM) to provide health care workers with continuous cardiovascular data and give patients insights into and promote their PA in daily life.

## Methods

### Study Population

Patients were included based on their diagnosis to form 3 patient groups: patients with HFrEF, patients with stable CAD and LVEF, and RAs who have visited a sports cardiologist before. Stable CAD is defined as the presence of angina pectoris caused by one or more coronary artery stenosis, which previously resulted in an acute coronary syndrome and required intervention (coronary artery bypass grafting, percutaneous coronary intervention, or medical therapy). The condition is considered stable when symptoms have remained unchanged in frequency, severity, and duration over time [21]. RAs were defined as men or women, >35 years of age, who perform sports at least 30 weeks a year, with a minimum of 2.5 hours of the same sport or 1.5 hours of different sports each week [22]. All 3 groups were analyzed separately. Patients were recruited via their cardiologist in the outpatient clinic of the Máxima Medical Center, the Netherlands. Eligible patients were contacted by the principal investigator, who provided verbal and written information about the validation study. Patients were excluded from the study if they had permanent atrial fibrillation, hemodynamically significant valvular disease, neurological or orthopedic conditions impairing physical exercise capacity, severe pulmonary disease impairing exercise capacity, peripheral vascular disease, or cognitive impairment. Patients had to be able to speak Dutch to be included.

### Protocol

Patients completed a laboratory activity protocol consisting of daily household activities reflecting real-life situations (cooking, table cleaning, and vacuuming), walking on a treadmill, and cycling. All activities were low-to-moderate intensity. The activity protocol was based on 2 similar studies in patients with cardiovascular disease, where it appeared to be functional and feasible in these patient groups [18,23]. The protocol was adjusted based on the patient population. Activity intensities were the highest for RAs, since they are in good condition and used to sport at higher intensities, while they were lower for patients with CAD and the lowest for patients with HFrEF. Cycling was done on 3 different loads, while walking was done at 3 different speeds and incline angles, all depending on the different patient groups. The duration of the entire protocol was around one hour. An overview of the protocol is shown in Table 1. The protocol was performed at the physical therapy department in the Máxima Medical Center under the supervision of a medical doctor.

**Table 1.** Activity protocol.

Activity type and activity	Duration (min)	Resting (min)
Sedentary activities		
Sitting	5	—^a^
Standing	2	—
Household activities		
Cooking	3	1
Cleaning	3	1
Vacuuming	3	3
Cycling (ergometer), load		
HFrEF^b^ 0 W; CAD^c^ 0 W; RA^d^ 0 W	3	3
HFrEF 25 W; CAD 40 W; RA 50 W	3	3
HFrEF 50 W; CAD 70 W; RA 100 W	3	3
Walking (treadmill), speed-incline		
HFrEF 2 km/h; CAD 4 km/h; RA 4 km/h-5%	3	3
HFrEF 4 km/h; CAD 5.5 km/h; RA 5.5 km/h-5%	3	3
HFrEF 2 km/h–5%; CAD 4 km/h–5%; RA 4 km/h–10%	3	3
Stairs		
Ascending	1	1
Descending	1	1

a Not applicable.

b HFrEF: heart failure with reduced ejection fraction.

c CAD: coronary artery disease.

d RA: recreational athlete.

### Criterion Measure

A CareFusion Oxycon Mobile (OM) device was used during the entire protocol to measure breath-by-breath oxygen (VO_2_) uptake and carbon dioxide (VCO_2_) production. This is a lightweight mobile device consisting of a facemask with a gas analyzer and a 12-lead electrocardiogram (ECG) sensor. The 12-lead ECG sensor was attached to the gas analyzer unit and strapped on a backpack, worn by the patient. The OM was connected to a computer where real-time data was gathered. Gas and volume calibration and ambient conditions were verified before the start of the protocol. The OM provides a reliable criterion measure as it has been validated before by comparing it with the gold standard of EE measurements, the Douglas Bag [24].

### Device

The Philips Health Band (PHB) is a Conformité Européenne–marked medical class IIa, wrist-worn device that measures and tracks movement and physiological parameters of the wearer. The PHB consists of different sensors, including a photoplethysmography sensor, an altimeter, and a tri-axial accelerometer. HR can be assessed through the photoplethysmography signal, while EE is estimated by an algorithm including basal metabolic rate (based on the wearer’s gender, age, height, and weight), activity, and HR. Patients wore the PHB on their nondominant wrist. The PHB was connected to the Philips Actigraphy Server System. The Philips Actigraphy Server System includes a mobile phone app and a Philips Health Suite Data Platform, where the data can be viewed and extracted by the authorized clinician. The Philips Actigraphy Server System was supplied with the most recent firmware updates.

### Data Analysis

Raw data from the breathing and HR analysis of the OM (sample rate 0.5 Hz) and the processed HR and EE data of the PHB (sample rate 0.0167 Hz) were exported and imported into a custom-made MATLAB (MATrix LABoratory; MathWorks) analysis program (R2023b [23.2.0.2409890]). The entire activity bounds were analyzed.

First, the EE was calculated from the OM breath-by-breath measurements using the Weir equation [25]:


$$EE=\left[\left(3.941\times VO_{2}\right)+\left(1.11\times VCO_{2}\right)\right]\times 1.1440$$

Outliers (eg, abrupt movements) in the HR and EE data were detected using a Hampel filter. Values exceeding 3 SDs from the median, calculated over the data point itself and up to 3 neighboring elements, were considered outliers and replaced with the median of this local window [26]. Afterward, the OM data were down-sampled to 0.0167 Hz to enable a correct comparison between the PHB data and the OM data. Then, the HR and EE data of the criterion measure (OM) and the device (PHB) were matched according to the timestamps corresponding to the activities of the protocol, as represented in Table 1, and were ready for comparison.

### Statistical Analysis

To achieve 80% power to detect an ICC of 0.75 (hypothesis 0), which is considered to indicate excellent agreement, a sample size of 19 participants per study group was calculated. This applies under the alternative hypothesis that an ICC of 0.4 (hypothesis 1) corresponds to poor agreement in the groups HFrEF, CAD, and RA.

Descriptive statistics were used to describe the population according to baseline clinical characteristics. Normality of the data was assessed by visual inspection of histograms and by interpreting skewness and kurtosis [27]. Between‐group differences (HFrEF vs CAD vs RA) were tested by one‐way ANOVA for continuous variables (age and LVEF), with Bonferroni-corrected post hoc comparisons where appropriate, and by Chi-square tests for categorical variables (sex and medication use). A 2-sided *P*<.05 was considered significant. The accuracy of the PHB was assessed by calculating the mean (SD), mean differences, and mean average percentage error (MAPE) in HR and EE obtained from the PHB compared with the criterion measure, the OM. These values were calculated per activity and over the entire protocol, including resting time.

One-sample *t* tests were performed using mean differences (between the PHB and the OM) compared with zero (hypothesis 0) to identify agreement between the PHB and the criterion measure within reasonable limits (set at a 10% error zone). In addition, Bland-Altman plots were created to illustrate the level of agreement between the estimated HR and EE, and the HR and EE from the criterion measure, with mean bias and 95% upper and lower limits of agreement (LoA). Data falling outside the LoA were inspected but did not meet any predefined exclusion criteria, such as extreme physiological values, poor signal quality, or documented device malfunctions. While there may be systematic errors under specific conditions (eg, high-intensity activities), these data were retained to ensure the analysis reflects the full range of real-world conditions encountered in the dataset.

To assess the reliability of the PHB for each activity and the entire protocol, the ICC using 2-way mixed models with absolute agreement was used. The ICC was considered poor below 0.4, fair between 0.4 and 0.59, good between 0.6 and 0.74, and excellent above 0.75 [28]. The responsiveness of the OM and PHB was assessed using a paired *t* test during cycling at different speeds and walking at different speeds and incline angles. All data analyses were performed using MATLAB (R2023b [23.2.0.2409890]).

### Ethical Considerations

Written informed consent was provided by all patients after they had received both oral and written information about the study. The validation study was approved by the local medical ethical committee of the Máxima Medical Center (institutional review board approval number NL79217.015.21) and was conducted in accordance with the Declaration of Helsinki. Patients did not receive any form of financial or material compensation for their participation.

## Results

### Patient Characteristics

A total of 57 patients were included and completed the activity protocol. The patients were equally divided into 3 groups: patients with HFrEF (n=19, mean age 69.5 years, SD 9.3 years), patients with CAD (n=19, mean age 63.7 years, SD 8.1 years), and RAs (n=19, mean age 58.8 years, SD 10.7 years). There was a significant difference in age across groups (ANOVA *P*=.004) and in LVEF (ANOVA *P*<.001), whereas gender distribution did not differ significantly (*χ*²_2_ *P*=.35). Patients across all groups were predominantly male, except for one female in the HFrEF group and 2 in the CAD group. The majority of HFrEF and CAD patients were using drugs affecting HR (19/19 HFrEF patients, 100%; 18/19 CAD patients, 95%), compared to only 5 in the RA group (26%). Between‐group differences were significant for β-blocker use (*χ*²_2_ *P*<.001) and amiodarone use (*χ*²_2_ *P*=.04), but not for calcium channel blockers (*P*=.11) or ivabradine (*P*=.36). Additional patient characteristics can be found in Table 2.

**Table 2.** Patient characteristics.

Variables	HF^a^ (n=19)	CAD^b^ (n=19)	RA^c^ (n=19)	*P* value
Age (years), mean (SD)	69.5 (9.3)	63.7 (8.1)	58.8 (10.7)	.004
Sex, n/N (%)				
Male	18/19 (95)	17/19 (90)	19/19 (100)	.35
Female	1/19 (5)	2/19 (11)	0	
LVEF^d^ (%), mean (SD)	37.7 (7.5)	58.8 (6.5)	61.8 (3.6)	<.001
HF etiology, n/N (%)				
iCMP^e^	9/19 (47)	—^f^	—	—
Non-iCMP	10/19 (53)	—	—	—
Medication, n/N (%)				
Beta-blocker	17/19 (90)	11/19 (58)	2/19 (11)	<.001
Calcium channel blocker	2/19 (11)	7/19 (37)	3/19 (16)	.11
Amiodarone	3/19 (16)	0	0	.04
Ivabradine	1/19 (5)	0	0	.36

a HF: heart failure.

b CAD: coronary artery disease.

c RA: recreational athlete.

d LVEF: left ventricular ejection fraction.

e iCMP: ischemic cardiomyopathy.

f Not applicable.

All data from the PHB of one RA was lost due to a synchronization problem and all data from the OM of one patient with HFrEF was lost due to technical problems, which resulted in the exclusion of these 2 patients from the validation analysis. Stair walking activities of 5 RAs, 5 patients with CAD, and 2 patients with HFrEF were excluded from the analysis due to OM measurement failure during that specific activity.

### Accuracy

#### Patients With HFrEF

Table S1 in Multimedia Appendix 1 illustrates the accuracy of HR and EE measurements by the PHB for patients with HFrEF.

**Figure 1.**
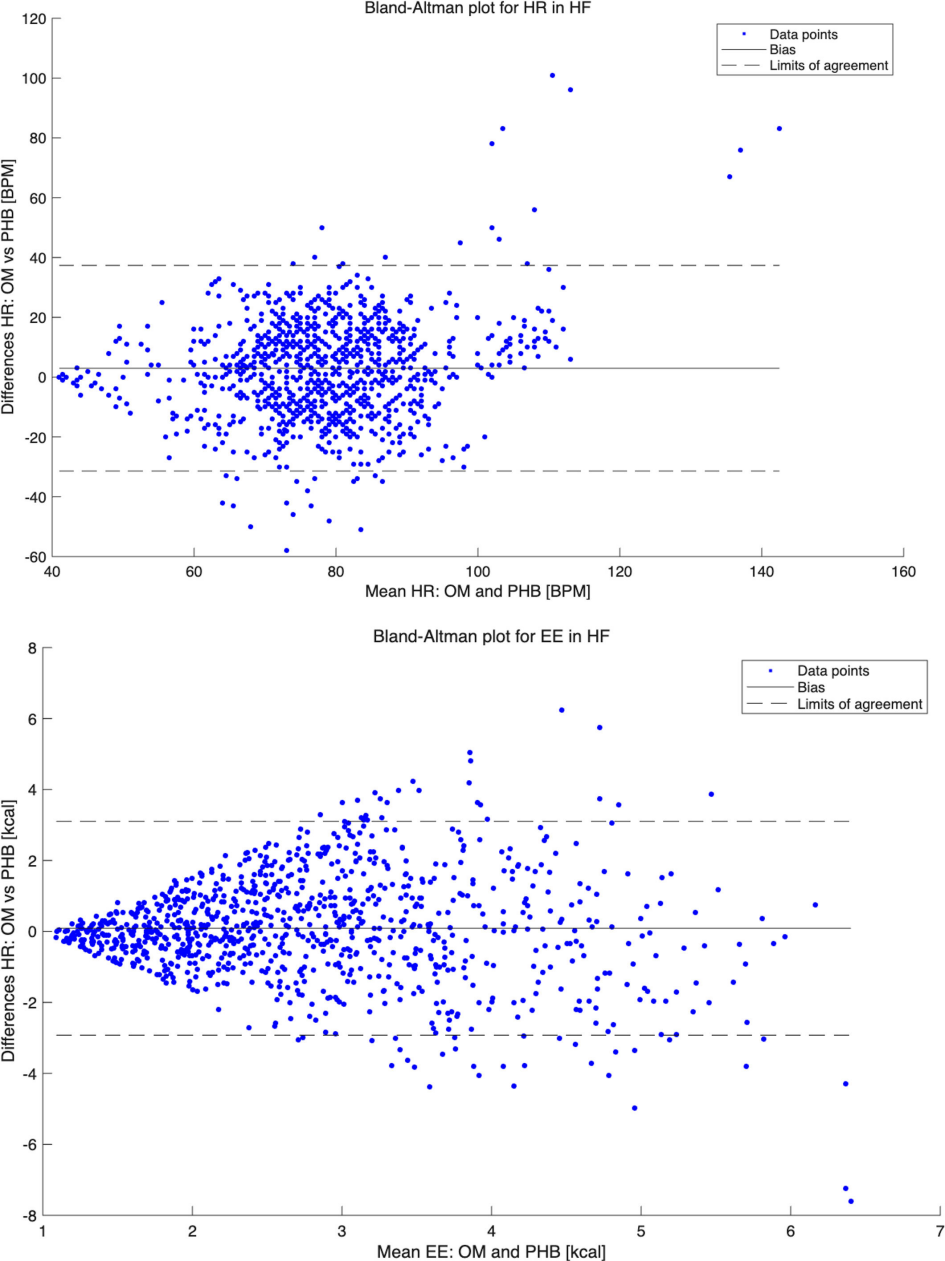
Bland-Altman plots heart rate and energy expenditure in patients with heart failure with reduced ejection fraction. BPM: beats per minute; EE: energy expenditure; HF: heart failure; HR: heart rate; OM: Oxycon Mobile; PHB: Philips Health Band.

For HR, the mean (SD) over the entire protocol in the HFrEF group was 80.0 (16.9) beats per minute (bpm) for OM and 77.1 (13.6) bpm for PHB. The PHB significantly underestimated HR over the entire protocol, with a mean difference of 3.0 bpm (*P*<.001), showing a similar underestimation pattern for moderate-intensity household activities, cycling, and walking. For resting and low-intensity household activities, there were no significant differences between HR assessed by PHB and OM, except for standing, showing an underestimation of 3.2 bpm (*P*<.05). Bland-Altman plots for total HR measurements showed the PHB’s underestimation, with wide LoA (lower LoA −30.7 bpm, upper LoA 36.7 bpm) (Figure 1). The bias was smaller for resting values (eg, for sitting, lower LoA −27.1 bpm, upper LoA 29.3 bpm) and increased with higher HR levels (eg, for cycling at 70 W, lower LoA −23.7 bpm, upper LoA 42.1 bpm). The ICCs for the total protocol indicated poor reliability, with a value of 0.36. The MAPE (SD) was 16.6 (13.9).

The EE results for the HFrEF group demonstrated a mean (SD) over the entire protocol of 2.86 (1.24) kcal for OM, and 2.76 (1.35) kcal for PHB (mean difference: 0.09 kcal, *P*=.06). However, significant underestimations were observed during climbing and walking down the stairs and cycling at 50 W, with mean differences of 0.54 kcal (*P*<.05), 1.04 kcal (*P*<.05), and 0.67 kcal (*P*<.001), respectively. It is important to note that resting and low-intensity household activities showed nonsignificant overestimations of EE, in contrast to other activities that were underestimated. Bland-Altman plots for total EE measurements indicated an underestimation by PHB, with a wide LoA for the total protocol (lower LoA −2.86 kcal, upper LoA 3.04 kcal; Figure 1). The bias for resting values was smaller (eg, for sitting, lower LoA −1.08 kcal, upper LoA 1.0 kcal), but increased toward EE values around 3 kcal, then stagnated (eg, cycling at 50 W, lower LoA −2.18, upper LoA 3.04). The ICCs for the total protocol indicated poor reliability, with a value of 0.32. The MAPE (SD) was 41.07 (40.53).

#### Patients With CAD

Table S2 of Multimedia Appendix 1 demonstrates the accuracy of HR and EE measurements by the PHB for patients with CAD.

**Figure 2.**
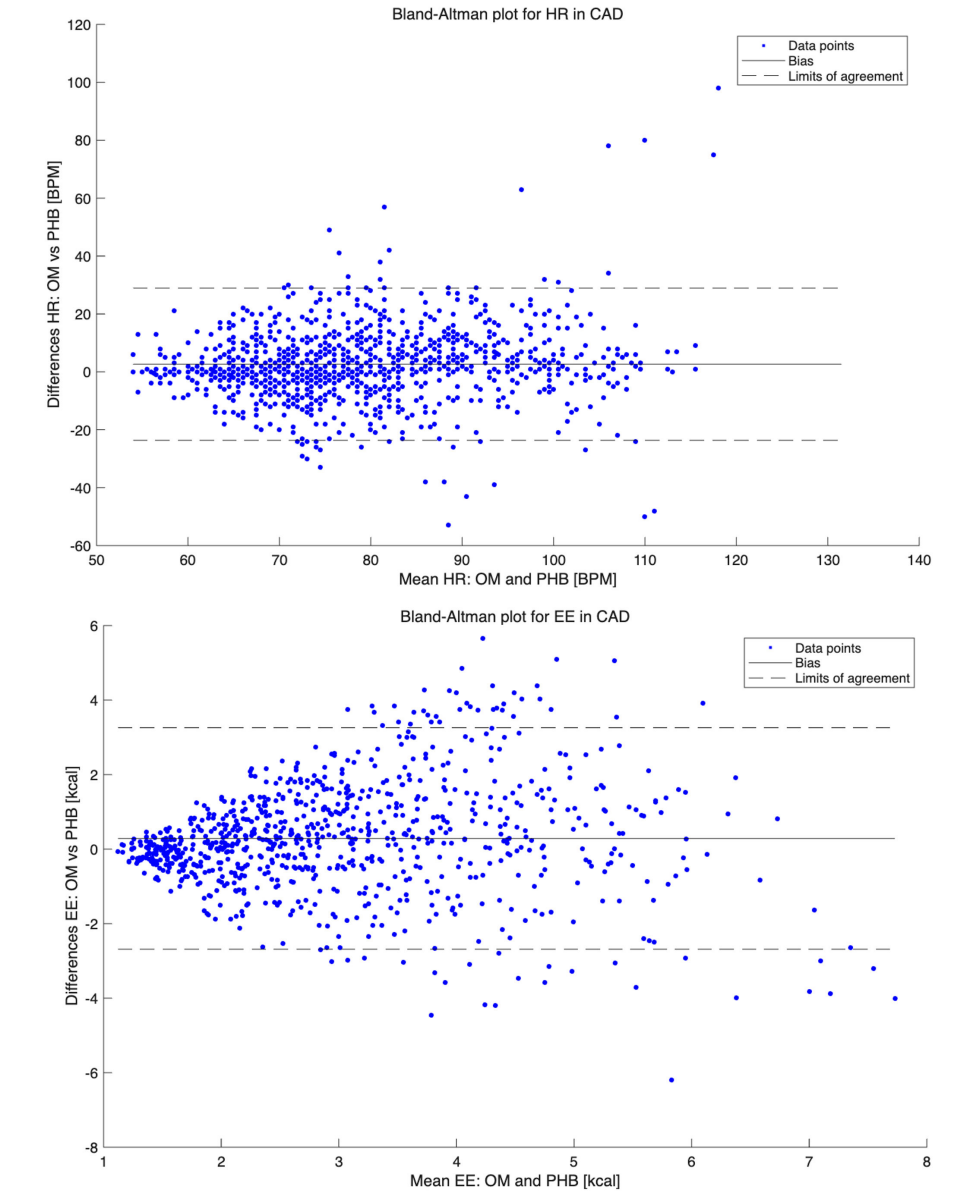
Bland-Altman plots for heart rate and energy expenditure in patients with coronary artery disease. BPM: beats per minute; CAD: coronary artery disease; EE: energy expenditure; HR: heart rate; OM: Oxycon Mobile; PHB: Philips Health Band.

For HR, the mean (SD) over the entire protocol in the CAD group was 80.4 (15.2) bpm for OM and 77.7 (13.3) bpm for PHB. The PHB significantly underestimated HR over the entire protocol, with a mean difference of 2.7 bpm (*P*<.001), showing a similar underestimation pattern across all activities except for sitting, cleaning the table, and cycling at 0 W, with mean differences of 0.9 bpm, 1.7 bpm, and 0.8 bpm, respectively (*P*>.05). Bland-Altman plots for total HR measurements illustrated the PHB’s underestimation, with a medium wide LoA (Figure 2). The PHB exhibited LoA from −23.1 bpm to 28.4 bpm. The bias was smaller for resting values (eg, for sitting, lower LoA −13.8 bpm, upper LoA 15.5 bpm) and increased with higher HR levels (eg, for cycling at 70 W, lower LoA −29.3 bpm, upper LoA 49.0 bpm). The ICCs for the total protocol indicated fair reliability, with a value of 0.55. The MAPE (SD) was 10.8 (10.7).

The EE results for the CAD group demonstrated a mean (SD) over the entire protocol of 3.16 (1.48) kcal for OM and 2.88 (1.41) kcal for PHB. The PHB significantly underestimated EE across the entire protocol, with a mean difference of 0.29 kcal (*P*<.001). A similar underestimation pattern was observed for moderate-intensity household activities (except for climbing the stairs) and walking (except for walking at 4 km/h). For resting, lower intensity household activities, and cycling, the PHB showed nonsignificant differences compared to OM. Bland-Altman plots for total EE measurements indicated an underestimation by PHB, with wide LoA for higher EE values and narrow LoA for lower EE values (Figure 2). The PHB exhibited LoA from −2.63 kcal to 3.20 kcal for the total protocol. The bias for resting values was small (eg, for sitting, lower LoA −0.74 kcal, upper LoA 0.84 kcal) and increased with higher EE levels (eg, cycling at 70 W, lower LoA −3.71 kcal, upper LoA 4.09 kcal). The ICCs for the total protocol revealed fair reliability, with a value of 0.46. The MAPE (SD) was 35.66 (34.83).

#### Recreational Athletes

Table S3 of Multimedia Appendix 1 demonstrates the accuracy of HR and EE measurements by the PHB for RAs.

**Figure 3.**
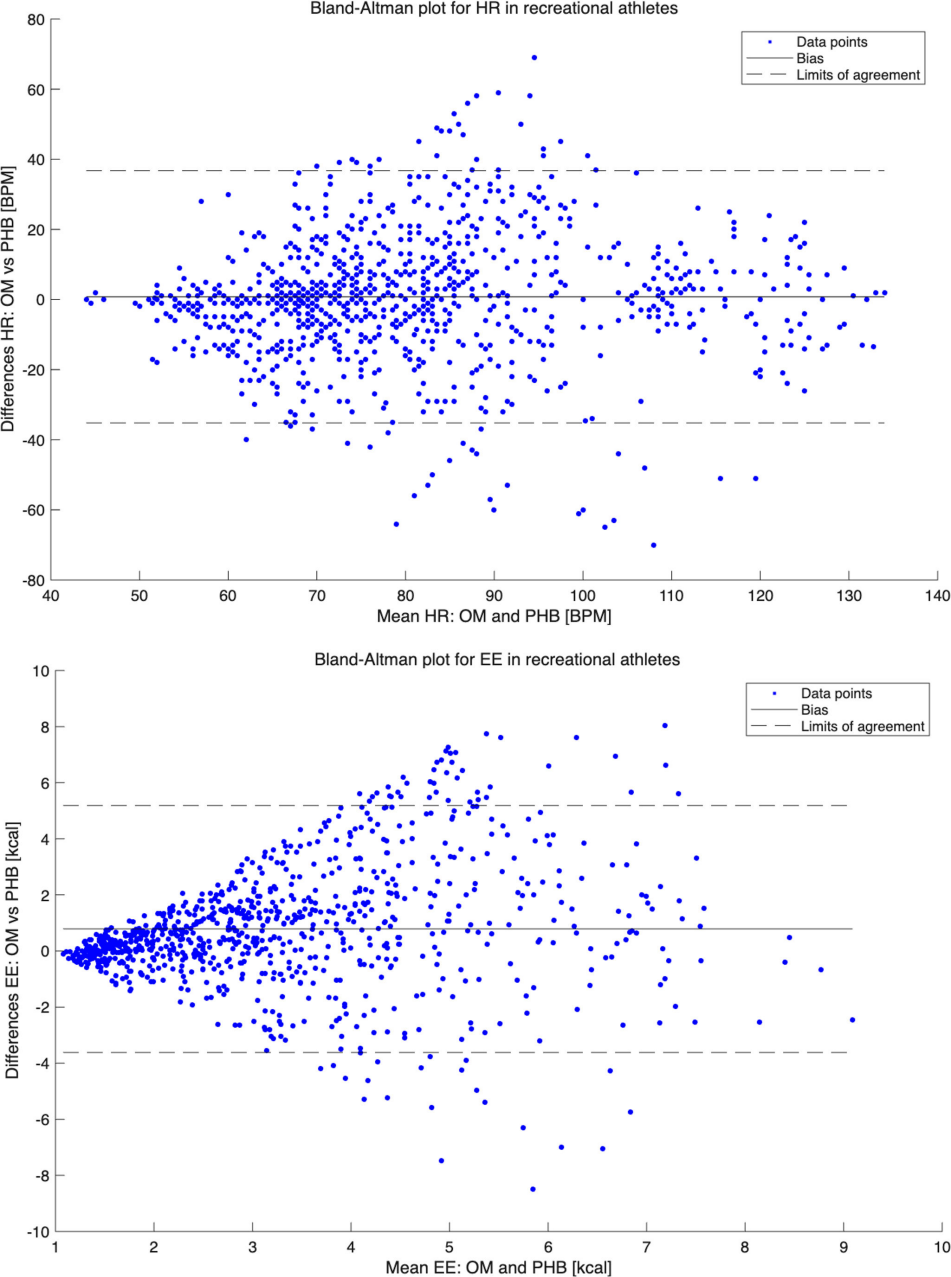
Bland-Altman plots for heart rate and energy expenditure in recreational athletes. BPM: beats per minute; EE: energy expenditure; HR: heart rate; OM: Oxycon Mobile; PHB: Philips Health Band.

For HR, the mean (SD) over the entire protocol in the RA group was 81.0 (20.8) bpm for OM and 80.2 (19.5) bpm for PHB. The PHB showed nonsignificant (*P*>.05) underestimations over the entire protocol, with a mean difference of 0.8 bpm. Significant underestimations were found only for walking at all speeds, cycling (except at 0 W), and standing. For the other activities, there were nonsignificant (*P*>.05) differences between HR measurements by OM and PHB. Bland-Altman plots for total HR measurements illustrated the PHB’s underestimation, with LoA from −34.5 bpm to 36.0 bpm (Figure 3). The bias was smaller for resting values (eg, for sitting, lower LoA −14.1 bpm, upper LoA 13.6 bpm) and increased with higher HR levels (eg, for cycling at 70 W, lower LoA −22.3 bpm, upper LoA 44.0 bpm). The ICCs for the total protocol indicated good reliability, with a value of 0.60. The MAPE (SD) was 16.2 (17.2).

The EE results for RAs demonstrated a mean (SD) over the entire protocol of 3.80 (SD 2.11) kcal for OM and 2.96 (SD 1.71) kcal for PHB. The PHB significantly underestimated EE across the entire protocol, with a mean difference of 0.79 kcal (*P*<.001). This underestimation pattern was consistent across most activities, with only nonsignificant (*P*>.05) underestimations for standing (mean difference of 0.08 kcal), cooking (mean difference of 0.04 kcal), and cycling at 0 W (mean difference of 0.18 kcal). Bland-Altman plots for total EE measurements indicated an underestimation by PHB, with wide LoA for higher EE values and narrower LoA for lower EE values (Figure 3). The bias increased until EE expenditures were around 5 kcal and then decreased. The PHB exhibited LoA from −3.53 kcal to 5.10 kcal for the total protocol. The bias for resting values was small (eg, for sitting, lower LoA −0.93 kcal, upper LoA 1.55 kcal) and increased with higher EE levels (eg, cycling at 100 W, lower LoA −3.66 kcal, upper LoA 6.41 kcal). The ICCs for the total protocol revealed poor reliability, with a value of 0.26. The MAPE (SD) was 42.87 (38.51).

### Responsiveness

Table S4 of Multimedia Appendix 1 shows the ability of PHB to detect within-patient changes in cycling and walking activities.

#### Patients With HFrEF

For HR responses, the PHB was able to detect within-patient changes when cycling at 25 W versus 50 W (mean difference −1.28 bpm; *P*=.02) and when walking at 2 km/h versus 4 km/h (mean difference −3.78 bpm; *P*=.009). Note that differences in HR between cycling at 0 W versus 25 W were nonsignificant as measured by the OM. For EE responses, the PHB detected within-patient changes for cycling at 0 W versus 50 W (mean difference −0.44 kcal; *P*<0.001) and at 25 W versus 50 W (mean difference −0.42 kcal; *P*<.001). However, the PHB was not able to detect within-patient changes in EE for walking. It should be noted that differences in EE were nonsignificant for cycling at 0 W versus 25 W and for walking at 2 km/h versus 2 km/h with a 5% slope, as was measured by the OM.

#### Patients With CAD

For HR responses, the PHB was able to detect within-patient changes when cycling at 0 W versus 70 W (mean difference −4.32 bpm; *P*=.003) and when cycling at 40 W versus 70 W (mean difference −3.76 bpm; *P*<.001). For walking, the PHB was able to detect within-patient changes when walking at 4 km/h versus walking at 4 km/h with a 5% slope (mean difference −4.26 bpm; *P*<.001). Note that there were no significant differences between walking at 5.5 km/h versus walking at 4 km/h with a 5% slope, as measured by the OM. For EE responses, the PHB can detect within-patient changes when cycling at 0 W versus 70 W (mean difference −0.74 kcal; *P*<.001) and when cycling at 40 W versus 70 W (mean difference −0.57 kcal; *P*<.001). However, the PHB was not able to detect within-patient changes in EE for walking at different speeds and slopes. It should be noted that there were no significant changes in EE when walking at 5.5 km/h versus walking at 4 km/h with a 5% slope, as measured by the OM.

#### Recreational Athletes

For HR responses, the PHB was able to detect within-patient changes for cycling and walking at different watts, speeds, and slopes, except when cycling at 0 W versus 50 W. However, no significant differences were present when walking at 5.5 km/h with a 5% slope versus walking at 4 km/h with a 10% slope. For EE responses, the PHB detected within-patient changes for cycling and walking at different watts, speeds, and slopes, except for walking at 4 km/h with a 5% slope versus walking at 5.5 km/h with a 5% slope. There were no significant differences between walking at 5.5 km/h with a 5% slope versus walking at 4 km/h with a 10% slope, as measured by the OM.

## Discussion

### Principal Findings

This validation trial demonstrated poor accuracy of the PHB for monitoring HR in patients with HFrEF and patients with CAD, while there was no significant difference between the PHB and OM in the RA group, showing its ability to correctly measure HR in a healthier population. For all 3 groups, there was a pattern of underestimating HR and EE during more intense activities. EE was significantly underestimated in patients with CAD and RAs over the entire protocol. Responsiveness of the PHB demonstrated mixed results. The PHB was able to detect within-patient changes in HR and EE in RAs for almost all cycling loads and walking speeds. In patients with CAD and patients with HFrEF, the PHB demonstrated moderate to poor responsiveness to changes in cycling loads or walking speeds.

### Accuracy

Our study showed that the PHB demonstrates poor accuracy for measuring HR in patients with HFrEF and patients with CAD during moderate intensity activities. This is in contrast to previous studies investigating commercially available wrist-worn photoplethysmography sensors. Blok et al [29] investigated the accuracy of heartbeat detection using photoplethysmography sensors in patients with cardiovascular disease. They concluded that photoplethysmography sensors can determine HR with high accuracy in patients with cardiovascular disease. However, these measurements were made in the resting state. During activities, photoplethysmography signals are often contaminated by motion artifacts and noise, which deteriorate the signal quality and pose significant challenges on HR monitoring. This has led to different research suggesting algorithms for accurate HR tracking even in the presence of motion artifacts and noises [30]. Novel photoplethysmography-based sensors are integrated with algorithms for HR estimation even during activities. Kim et al [31] validated 2 new commercially available smartwatches for the assessment of HR during a cardiopulmonary exercise test in patients with CAD. They concluded that these newer devices show high concordance with the gold-standard ECG measurement. These results are also in contrast to the findings from our validation trial. A possible explanation for this might be the difference in the activity protocol. While Kim et al [31] validated the photoplethysmography sensors during a cardiopulmonary exercise test, we tried to validate the PHB sensor during an activity protocol with household activities reflecting real-life situations. These household activities included cooking, table cleaning, and vacuuming, which require more wrist movements. The placement of the photoplethysmography sensor on different body parts affects the severity of motion artifacts. Wrist placement is convenient since the photoplethysmography sensor can be integrated into smartwatches and fitness trackers, but the wrist is more prone to motion artifacts and sensor detachment due to hand movement [32]. Moreover, the skin on the wrist moves more than other body parts, affecting sensor stability, likely influencing signal quality [32]. Conversely, placing the photoplethysmography sensor higher on the underarm or on the upper arm could reduce motion artifacts, since these areas experience less motion during daily activities, and skin movement is minimal compared to the wrist.

Another finding demonstrates a significant difference in the accuracy of HR between patients with HFrEF and patients with CAD compared to the . A possible explanation for this difference might be the patient’s medication use and their cardiovascular pathology. Almost all patients in both HFrEF and CAD groups used drugs affecting their HR. In the RA group, only 26% of participants used drugs affecting HR. In addition, patients with HFrEF often endure chronotropic incompetence, which might affect HR estimation by the algorithm analyzing the photoplethysmography signal. The mechanism behind this in cardiovascular patients is that photoplethysmography-based HR measurement algorithms rely on detecting pulsatile blood volume changes in the peripheral microvasculature, which are often attenuated in these patients by reduced stroke volume and peripheral vasoconstriction. This leads to lower signal amplitude and distorted waveform morphology that can cause missed beats or misidentified peaks [33]. Chronotropic incompetence further narrows the dynamic range of HR changes, challenging algorithms tuned to larger beat-to-beat interval variability [33]. Blunting of the systolic upstroke by β-blockers and other rate-controlling medications alters the temporal features critical for peak detection. Moreover, other conditions like valvular heart disease or peripheral artery disease may similarly impair photoplethysmography signal quality due to altered vascular compliance or flow characteristics [34]. This stresses the need for more patient-specific algorithms for assessing HR through photoplethysmography signals. Potential pathways include dynamic peak detection thresholds, signal quality indexing, or machine-learning models trained on data from cardiovascular populations. These models could be tailored based on known patient characteristics (eg, presence of heart failure and medication use) and integrated into device firmware [35].

Our findings demonstrate a statistically significant difference in HR measurements between the PHB sensor and the OM in both HFrEF and CAD groups. However, the mean difference of approximately 3 bpm across the entire activity protocol is relatively small, raising questions about the clinical relevance of this discrepancy. Nonetheless, our data also demonstrates that the bias in HR measurements increases with rising HR values. This indicates that the measurement error becomes more pronounced at higher intensities, potentially leading to clinically meaningful discrepancies, particularly in contexts where the PHB sensor is used to support clinical decision-making, such as in RPM or cardiac telerehabilitation.

Our trial demonstrated that the PHB significantly underestimated EE over the entire protocol for patients with CAD and RAs. Gemini et al [20] conducted a systematic review examining studies that investigated the accuracy and acceptability of commercially available smartwatches. Of the 24 included studies, 22 assessed PA using EE as the outcome measure. Overall, all sensors demonstrated a MAPE of over 30%, indicating poor accuracy across all devices for assessing EE. The underestimation of EE by noninvasive sensors has also been observed in other studies. This aligns with our findings. All three groups showed an increase in underestimation with increasing activity intensity. This is in contrast to the findings from Herkert et al [18], who investigated 2 commercially available activity trackers in patients with CAD and patients with HFrEF. They observed an overestimation of EE over the entire protocol and an increase in overestimation when the activities intensify. This difference may possibly be explained by the variation in algorithms used to estimate EE. An alternative explanation for the underestimation of EE and its increase with intensified activities possibly lies within the HR sensor. Most algorithms for predicting EE in wrist-worn sensors are based on HR and accelerometer measurements. During this trial, we observed that the PHB significantly underestimated HR in patients with HFrEF and patients with CAD. Since these HR measurements are used to predict EE during these activities, it is expected that the underestimation would also be reflected in the EE prediction. Another explanation could be the simulation of the use of walking aids, which restrict arm movement in patients, by holding the handlebars of the treadmill. This restriction leads to decreased accelerometer measurements, resulting in a lower prediction of EE during those activities.

### Responsiveness

The PHB was able to detect some changes in both walking and cycling loads in patients with HFrEF and patients with CAD. However, the responsiveness of the PHB in RAs was a lot better compared to HFrEF and CAD groups. Research investigating the responsiveness of wrist-worn devices is scarce, especially in patients with cardiovascular disease; almost all trials focus their research solely on accuracy. Responsiveness is an important feature of smart devices for monitoring exercise activities at home. Herkert et al [18] investigated the responsiveness of 2 commercially available wrist-worn devices in patients with HFrEF and patients with CAD. They concluded that both sensors showed poor performance in detecting within-patient changes in the low-to-moderate exercise intensity domain. These findings are confirmed by our validation trial. Even though the PHB showed better responsiveness, there is still a lot of room for improvement, stressing the need for better algorithms for detecting within-patient changes during exercises for patients with cardiovascular disease.

### Future Perspectives

Our study clearly shows that even measurements of medically certified devices, using photoplethysmography and accelerometer to assess HR and EE, should be interpreted with caution for patients with cardiovascular disease. More studies with patients with cardiovascular disease and noninvasive sensors, using photoplethysmography and accelerometer, for assessing HR and EE should be done to enhance algorithm development. It is crucial that these trials extract raw photoplethysmography and accelerometer signals for better algorithm development. In addition, it is important that the validation of these new algorithms is conducted using an activity protocol that reflects the patients’ daily lives, rather than solely during exercise tests or rest measurements. Furthermore, future validation studies should not only focus on accuracy but also on the responsiveness of the sensors, as this is crucial for detecting within-patient changes throughout the day. Finally, to address existing barriers that hinder the usage of mHealth solutions and to assist health care professionals in evaluating the level of available evidence, a task force initiated by the European Society of Cardiology regulatory affairs committee formulated both general and specific criteria through a consensus process. These criteria should be consulted before considering the implementation of noninvasive devices in health care settings to ensure patient safety [36].

### Strengths and Limitations

A strength of this trial is that both patients with chronic cardiovascular diseases and RAs are included. In the results, there is a significant difference between the accuracy of the PHB for RA and for patients with cardiovascular disease in both accuracy and responsiveness. Stressing the need for algorithms that take into account both the cardiovascular pathology of the patients and medication usage. A limitation of this trial is the fact that patients were tested in a laboratory setting, even though the activity protocol consists of activities reflecting patients’ daily life. This means that the results might not be able to be extrapolated to free-living conditions. In addition, a single activity protocol per patient group has been used to maximize the reproducibility of the study. Future validation trials could consider personalizing activity protocols, as cardiac patients show substantial variability in peak VO_2_ and anaerobic threshold. Another limitation lies within the patient population. The majority of patients were men, making it possible that these results are not applicable to women.

### Conclusion

HR and EE assessment of a medically certified noninvasive sensor using a photoplethysmography and accelerometer showed poor accuracy and moderate responsiveness during an activity protocol reflecting daily living activities in chronic cardiac patients (HFrEF and CAD). High accuracy was obtained for HR in RA, while responsiveness was acceptable. This research confirms previous research and stresses the need for better patient-specific algorithms, taking cardiovascular pathology and medication usage into account, for assessing HR and EE.

## Supplementary material

10.2196/69343 Multimedia Appendix 1 The appendix file contains the tables with the results from the statistical analysis.

## Abbreviations

bpm: beats per minute
CAD: coronary artery disease
CR: cardiac rehabilitation
ECG: electrocardiogram
EE: energy expenditure
HFrEF: heart failure with reduced ejection fraction
HR: heart rate
LoA: limits of agreement
LVEF: left ventricular ejection fraction
MAPE: mean average percentage error
OM: Oxycon Mobile
PA: physical activity
PHB: Philips Health Band
RA: recreational athlete
RPM: remote patient monitoring

## References

[1] Benjamin EJ, Muntner P, Alonso A (2019). Heart disease and stroke statistics-2019 update: a report from the American Heart Association. Circulation.

[2] Vooturi S, Anil P, Monica Y (2023). Effects of exercise training and physical activity in patients with coronary artery disease. Indian J Clin Cardiol.

[3] Sakai T, Yagishita A, Morise M (2021). Impact of exercise capacity on the long-term incidence of atrial arrhythmias in heart failure. Sci Rep.

[4] Myers J, Prakash M, Froelicher V, Do D, Partington S, Atwood JE (2002). Exercise capacity and mortality among men referred for exercise testing. N Engl J Med.

[5] Hansen D, Abreu A, Ambrosetti M (2022). Exercise intensity assessment and prescription in cardiovascular rehabilitation and beyond: why and how: a position statement from the secondary prevention and rehabilitation section of the European Association of Preventive Cardiology. Eur J Prev Cardiol.

[6] Taylor RS, Brown A, Ebrahim S (2004). Exercise-based rehabilitation for patients with coronary heart disease: systematic review and meta-analysis of randomized controlled trials. Am J Med.

[7] Corrà U, European Association of Cardiovascular Prevention and Rehabilitation Committee for Science Guidelines, EACPR (2010). Secondary prevention through cardiac rehabilitation: physical activity counselling and exercise training: key components of the position paper from the Cardiac Rehabilitation Section of the European Association of Cardiovascular Prevention and Rehabilitation. Eur Heart J.

[8] van Engen-Verheul M, de Vries H, Kemps H, Kraaijenhagen R, de Keizer N, Peek N (2013). Cardiac rehabilitation uptake and its determinants in the Netherlands. Eur J Prev Cardiol.

[9] Lin MH, Yuan WL, Huang TC, Zhang HF, Mai JT, Wang JF (2017). Clinical effectiveness of telemedicine for chronic heart failure: a systematic review and meta-analysis. J Investig Med.

[10] Zhu Y, Gu X, Xu C (2020). Effectiveness of telemedicine systems for adults with heart failure: a meta-analysis of randomized controlled trials. Heart Fail Rev.

[11] Koehler F, Koehler K, Deckwart O (2018). Efficacy of telemedical interventional management in patients with heart failure (TIM-HF2): a randomised, controlled, parallel-group, unmasked trial. Lancet.

[12] De Lathauwer ILJ, Nieuwenhuys WW, Hafkamp F (2025). Remote patient monitoring in heart failure: a comprehensive meta‐analysis of effective programme components for hospitalization and mortality reduction. Eur J Heart Fail.

[13] Menditto A, Patriarca M, Magnusson B (2007). Understanding the meaning of accuracy, trueness and precision. Accred Qual Assur.

[14] Wallen MP, Gomersall SR, Keating SE, Wisløff U, Coombes JS (2016). Accuracy of heart rate watches: implications for weight management. PLoS One.

[15] Chowdhury EA, Western MJ, Nightingale TE, Peacock OJ, Thompson D (2017). Assessment of laboratory and daily energy expenditure estimates from consumer multi-sensor physical activity monitors. PLoS ONE.

[16] Bai Y, Hibbing P, Mantis C, Welk GJ (2018). Comparative evaluation of heart rate-based monitors: Apple Watch vs Fitbit Charge HR. J Sports Sci.

[17] Shcherbina A, Mattsson CM, Waggott D (2017). Accuracy in wrist-worn, sensor-based measurements of heart rate and energy expenditure in a diverse cohort. J Pers Med.

[18] Herkert C, Kraal JJ, van Loon EMA, van Hooff M, Kemps HMC (2019). Usefulness of modern activity trackers for monitoring exercise behavior in chronic cardiac patients: validation study. JMIR mHealth uHealth.

[19] Falter M, Budts W, Goetschalckx K, Cornelissen V, Buys R (2019). Accuracy of Apple watch measurements for heart rate and energy expenditure in patients with cardiovascular disease: cross-sectional study. JMIR mHealth uHealth.

[20] Germini F, Noronha N, Borg Debono V (2022). Accuracy and acceptability of wrist-wearable activity-tracking devices: systematic review of the literature. J Med Internet Res.

[21] Montalescot G, Sechtem U, Task Force Members (2013). 2013 ESC guidelines on the management of stable coronary artery disease: the Task Force on the management of stable coronary artery disease of the European Society of Cardiology. Eur Heart J.

[22] Van Middelkoop M, Kolkman J, Van Ochten J, Bierma-Zeinstra SMA, Koes BW (2008). Risk factors for lower extremity injuries among male marathon runners. Scand J Med Sci Sports.

[23] Kraal JJ, Sartor F, Papini G (2016). Energy expenditure estimation in beta-blocker-medicated cardiac patients by combining heart rate and body movement data. Eur J Prev Cardiol.

[24] Rosdahl H, Gullstrand L, Salier-Eriksson J, Johansson P, Schantz P (2010). Evaluation of the Oxycon Mobile metabolic system against the Douglas bag method. Eur J Appl Physiol.

[25] WEIR J (1949). New methods for calculating metabolic rate with special reference to protein metabolism. J Physiol.

[26] Pearson RK (2002). Outliers in process modeling and identification. IEEE Trans Contr Syst Technol.

[27] Kim HY (2013). Statistical notes for clinical researchers: assessing normal distribution (2) using skewness and kurtosis. Restor Dent Endod.

[28] Cicchetti DV (1994). Guidelines, criteria, and rules of thumb for evaluating normed and standardized assessment instruments in psychology. Psychol Assess.

[29] Blok S, Piek MA, Tulevski II, Somsen GA, Winter MM (2021). The accuracy of heartbeat detection using photoplethysmography technology in cardiac patients. J Electrocardiol.

[30] Zhu L, Kan C, Du Y, Du D (2018). Heart rate monitoring during physical exercise from photoplethysmography using neural network. IEEE Sens Lett.

[31] Kim C, Song JH, Kim SH (2023). Validation of wearable digital devices for heart rate measurement during exercise test in patients with coronary artery disease. Ann Rehabil Med.

[32] Fine J, Branan KL, Rodriguez AJ (2021). Sources of inaccuracy in photoplethysmography for continuous cardiovascular monitoring. Biosensors (Basel).

[33] Mejía-Mejía E, Allen J, Budidha K, El-Hajj C, Kyriacou PA, Charlton PH, Kyriacou PA, Budidha K (2021). Photoplethysmography: Technology, Signal Analysis, and Applications.

[34] Allen J (2007). Photoplethysmography and its application in clinical physiological measurement. Physiol Meas.

[35] Zhang Z, Pi Z, Liu B (2015). TROIKA: a general framework for heart rate monitoring using wrist-type photoplethysmographic signals during intensive physical exercise. IEEE Trans Biomed Eng.

[36] Caiani EG, Kemps H, Hoogendoorn P (2024). Standardized assessment of evidence supporting the adoption of mobile health solutions: a clinical consensus statement of the ESC Regulatory Affairs Committee. Eur Heart J Digit Health.

