# Supplementary material for: Analysis of a Medically Certified, Wrist-Worn Sensor for the Assessment of Heart Rate and Energy Expenditure During Daily Activities in Patients With Chronic Heart Failure or Coronary Artery Disease and Recreational Athletes: Validation Study
Source: JMIR Cardio. 2025 Sep 30;9:e69343. doi: 10.2196/69343 (PMC12483477; doi:10.2196/69343)
Supplement: Multimedia Appendix 1 [file cardio-v9-e69343-s001.docx]

**Appendix validation trial**

*Table S1. Accuracy of HR and EE measurements by PHB for HFrEF patients*

| Heart Failure patients with reduced ejetion fraction | HR OM  (Criterion measure)  **Mean ± SD**  **[BPM]** | HR PHB  **Mean ± SD**  **[BPM]** | OM vs PHB | | | | | EE OM  (Criterion measure)  **Mean ± SD**  **[kcal]** | EE PHB  **Mean ± SD**  **[kcal]** | OM vs PHB | | | | |
| --- | --- | --- | --- | --- | --- | --- | --- | --- | --- | --- | --- | --- | --- | --- |
|  |  |  | **Mean difference**  **[BPM]** | **Lower LoA**  **[BPM]** | **Upper LoA**  **[BPM]** | **ICC** | **MAPE ± SD**  **[%]** |  |  | **Mean difference**  **[kcal]** | **Lower LoA**  **[kcal]** | **Upper LoA**  **[kcal]** | **ICC** | **MAPE ± SD**  **[%]** |
| *Resting* |  |  |  |  |  |  |  |  |  |  |  |  |  |  |
| - *Sitting* | 68.49±15.16 | 67.40±11.90 | 1.09 | -27.10 | 29.27 | 0.45 | 12.44±10.49 | 1.45±0.39 | 1.50±0.42 | -0.04 | -1.08 | 1.00 | 0.15 | 30.06±24.26 |
| - *standing* | 72.22±12.72 | 69.04±12.09 | 3.19* | -18.98 | 25.35 | 0.55 | 13.39±9.06 | 1.58±0.50 | 1.60±0.48 | -0.03 | -1.40 | 1.35 | 0.03 | 31.02±33.70 |
| *Low intensity household activities* |  |  |  |  |  |  |  |  |  |  |  |  |  |  |
| - *Cooking* | 79.51±15.81 | 75.63±12.75 | 3.72 | -29.42 | 36.86 | 0.28 | 15.85±14.57 | 2.16±1.03 | 2.71±1.32 | -0.48 | -3.52 | 2.57 | 0.12 | 73.19±69.15 |
| - *Cleaning the table* | 80.60±17.35 | 78.88±12.99 | 1.72 | -36.86 | 40.30 | 0.18 | 18.06±15.24 | 2.90±1.14 | 3.17±1.16 | -0.29 | -3.16 | 2.58 | 0.15 | 47.74±52.32 |
| *Moderate intensity household activities* |  |  |  |  |  |  |  |  |  |  |  |  |  |  |
| - *Vacuum cleaning* | 85.03±17.59 | 77.00±13.42 | 8.03** | -26.13 | 42.19 | 0.27 | 16.06±10.35 | 3.11±0.99 | 2.94±1.26 | 0.14 | -2.29 | 2.57 | 0.41 | 33.46±30.79 |
| - *Climbing the stairs* | 82.92±12.54 | 73.38±10.39 | 9.54* | -17.84 | 36.91 | 0.07 | 16.34±9.26 | 3.20±1.51 | 2.66±0.88 | 0.54* | -2.15 | 3.24 | 0.31 | 34.33±25.89 |
| - *Walking down the stairs* | 89.91±9.61 | 81.26±10.20 | 8.65* | -19.11 | 36.42 | 0.13 | 14.14±10.51 | 3.97±1.24 | 2.93±1.19 | 1.04* | -2.66 | 4.73 | 0.25 | 38.66±27.82 |
| *Cycling* |  |  |  |  |  |  |  |  |  |  |  |  |  |  |
| - *cycling at 0 w (70 rpm)* | 81.11±19.97 | 75.50±12.71 | 5.61* | -32.69 | 43.91 | 0.28 | 15.07±11.97 | 2.62±0.98 | 2.50±1.08 | 0.15 | -2.60 | 2.89 | 0.05 | 38.89±33.94 |
| - *cycling at 25 w (70 rpm)* | 81.11±21.85 | 75.17±14.34 | 5.94* | -30.87 | 42.75 | 0.43 | 17.02±10.31 | 2.83±0.99 | 2.56±1.16 | 0.26 | -2.22 | 2.73 | 0.27 | 38.17±26.60 |
| - *cycling at 50 w (70 rpm)* | 85.43±19.09 | 76.22±14.75 | 9.21** | -23.69 | 42.10 | 0.37 | 18.07±10.62 | 3.65±1.37 | 2.96±1.49 | 0.67** | -2.18 | 3.52 | 0.37 | 33.39±21.44 |
| *Walking* |  |  |  |  |  |  |  |  |  |  |  |  |  |  |
| - *walking at 2 km/h* | 81.13±13.53 | 76.92±13.05 | 4.21* | -25.76 | 34.18 | 0.30 | 15.74±13.05 | 3.13±0.98 | 3.08±1.71 | 0.07 | -3.59 | 3.73 | 0.10 | 47.20±47.15 |
| - *walking at 4 km/h* | 85.28±15.46 | 80.36±14.28 | 4.92* | -30.03 | 39.87 | 0.24 | 17.39±13.34 | 3.83±1.33 | 3.27±1.59 | 0.61* | -2.87 | 4.10 | 0.20 | 37.80±26.76 |
| - *walking at 2 km/h and 5% slope* | 82.56±14.58 | 79.22±12.95 | 3.34* | -26.38 | 33.06 | 0.37 | 15.62±15.17 | 3.29±1.19 | 3.01±1.55 | 0.30 | -3.19 | 3.79 | 0.15 | 43.84±32.75 |
| *Total protocol*  *(resting time included)* | 80.03±16.94 | 77.05±13.63 | 2.97** | -30.73 | 36.68 | 0.36 | 16.62±13.86 | 2.86±1.24 | 2.76±1.35 | 0.09 | -2.86 | 3.04 | 0.32 | 41.07±40.53 |

Mean differences are calculated as device minus criterion measure.

SD: Standard Deviation

LoA: Limits of Agreement

ICC: Intraclass correlation coefficient

MAPE: Mean Average Percentage Error

OM: Oxycon Mobile

PHB: Philips Health Band

BPM: Beats Per Minute

Kcal: Kilocalories

*p<0.05, **p<0.001

| Coronary Artery Disease patients | HR OM  (Criterion measure)  **Mean ± SD**  **[BPM]** | HR PHB  **Mean ± SD**  **[BPM]** | OM vs PHB | | | | | EE OM  (Criterion measure)  **Mean ± SD**  **[kcal]** | EE PHB  **Mean ± SD**  **[kcal]** | OM vs PHB | | | | |
| --- | --- | --- | --- | --- | --- | --- | --- | --- | --- | --- | --- | --- | --- | --- |
|  |  |  | **Mean difference**  **[BPM]** | **Lower LoA**  **[BPM]** | **Upper LoA**  **[BPM]** | **ICC** | **MAPE ± SD**  **[%]** |  |  | **Mean difference**  **[kcal]** | **Lower LoA**  **[kcal]** | **Upper LoA**  **[kcal]** | **ICC** | **MAPE ± SD**  **[%]** |
| *Resting* |  |  |  |  |  |  |  |  |  |  |  |  |  |  |
| - *Sitting* | 66.88±9.88 | 66.03±9.82 | 0.86 | -13.75 | 15.47 | 0.71 | 5.67±8.92 | 1.52±0.39 | 1.50±0.22 | 0.05 | -0.74 | 0.84 | 0.26 | 17.11±12.57 |
| - *standing* | 71.51±10.58 | 66.69±9.51 | 4.21** | -11.90 | 20.33 | 0.56 | 7.43±8.68 | 1.15±0.41 | 1.57±0.27 | 0.11 | -1.18 | 1.39 | 0.09 | 24.00±18.35 |
| *Low intensity household activities* |  |  |  |  |  |  |  |  |  |  |  |  |  |  |
| - *Cooking* | 75.15±10.40 | 72.63±10.82 | 2.53* | -13.43 | 18.49 | 0.67 | 8.16±7.33 | 2.02±0.64 | 2.54±1.41 | -0.42 | -3.02 | 2.19 | 0.26 | 42.11±44.69 |
| - *Cleaning the table* | 77.88±10.91 | 76.20±9.16 | 1.68 | -27.66 | 20.54 | 0.67 | 10.15±8.37 | 2.90±1.26 | 2.86±0.96 | 0.06 | -2.93 | 3.05 | 0.09 | 43.75±41.94 |
| *Moderate intensity household activities* |  |  |  |  |  |  |  |  |  |  |  |  |  |  |
| - *Vacuum cleaning* | 78.22±12.30 | 75.39±8.57 | 2.83* | -19.04 | 24.69 | 0.41 | 10.21±9.69 | 3.19±0.93 | 2.69±0.73 | 0.53** | -1.74 | 2.80 | 0.09 | 30.15±18.55 |
| - *Climbing the stairs* | 86.43±14.19 | 79.71±13.02 | 3.08* | -13.10 | 26.53 | 0.57 | 10.16±7.94 | 3.32±1.36 | 2.94±1.06 | 0.53 | -2.16 | 3.23 | 0.36 | 31.60±20.55 |
| - *Walking down the stairs* | 92.77±10.95 | 89.09±10.77 | 3.69* | -12.47 | 19.84 | 0.65 | 7.46±5.45 | 4.28±0.83 | 3.86±1.00 | 0.69* | -1.79 | 3.18 | 0.14 | 25.46±15.49 |
| *Cycling* |  |  |  |  |  |  |  |  |  |  |  |  |  |  |
| - *cycling at 0 w (70 rpm)* | 75.20±10.75 | 74.42±12.58 | 0.78 | -18.79 | 20.34 | 0.64 | 8.60±14.37 | 2.62±0.65 | 2.50±0.84 | 0.09 | -1.59 | 1.77 | 0.41 | 26.87±21.13 |
| - *cycling at 40 w (70 rpm)* | 82.33±19.50 | 74.97±12.48 | 7.36** | -28.20 | 42.91 | 0.30 | 11.24±11.89 | 3.03±1.26 | 3.01±1.31 | 0.08 | -2.79 | 2.95 | 0.37 | 38.08±29.98 |
| - *cycling at 70 w (70 rpm)* | 88.57±21.61 | 78.74±14.10 | 9.83** | -29.29 | 48.95 | 0.28 | 15.28±14.07 | 3.91±1.80 | 3.72±2.01 | 0.19 | -3.71 | 4.09 | 0.46 | 46.18±37.11 |
| *Walking* |  |  |  |  |  |  |  |  |  |  |  |  |  |  |
| - *walking at 4 km/h* | 89.84±13.32 | 77.28±12.02 | 6.07** | -21.72 | 33.85 | 0.28 | 13.02±11.74 | 3.65±1.40 | 3.22±1.43 | 0.41 | -2.95 | 3.78 | 0.26 | 41.64±41.41 |
| - *walking at 5.5 km/h* | 88.80±13.33 | 79.55±14.27 | 9.25** | -11.99 | 30.49 | 0.44 | 12.41±10.36 | 4.16±1.73 | 3.26±1.64 | 0.92** | -2.73 | 4.57 | 0.29 | 34.24±25.56 |
| - *walking at 4 km/h and 5% slope* | 87.84±13.75 | 82.16±11.38 | 5.68** | -12.59 | 23.34 | 0.59 | 9.53±6.80 | 4.12±1.58 | 3.37±1.68 | 0.78* | -2.82 | 4.37 | 0.30 | 36.75±27.41 |
| *Total protocol*  *(resting time included)* | 80.37±15.18 | 77.72±13.34 | 2.65** | -23.11 | 28.41 | 0.55 | 10.82±10.65 | 3.16±1.48 | 2.88±1.41 | 0.29** | -2.63 | 3.20 | 0.46 | 35.66±34.83 |

*Table S2. Accuracy of HR and EE measurements by PHB for CAD patients*

Mean differences are calculated as device minus criterion measure.

SD: Standard Deviation

LoA: Limits of Agreement

ICC: Intraclass correlation coefficient

MAPE: Mean Average Percentage Error

OM: Oxycon Mobile

PHB: Philips Health Band

BPM: Beats Per Minute

Kcal: Kilocalories

*p<0.05, **p<0.001

| Recreational Athletes | HR OM  (Criterion measure)  **Mean ± SD**  **[BPM]** | Heart rate  Philips Health band  **Mean ± SD**  **[BPM]** | OM vs PHB | | | | | EE OM  (Criterion measure)  **Mean ± SD**  **[kcal]** | EE PHB  **Mean ± SD**  **[kcal]** | OM vs PHB | | | | |
| --- | --- | --- | --- | --- | --- | --- | --- | --- | --- | --- | --- | --- | --- | --- |
|  |  |  | **Mean difference**  **[BPM]** | **Lower LoA**  **[BPM]** | **Upper LoA**  **[BPM]** | **ICC** | **MAPE ± SD**  **[%]** |  |  | **Mean difference**  **[kcal]** | **Lower LoA**  **[kcal]** | **Upper LoA**  **[kcal]** | **ICC** | **MAPE ± SD**  **[%]** |
| *Resting* |  |  |  |  |  |  |  |  |  |  |  |  |  |  |
| - *Sitting* | 65.30±15.00 | 65.56±13.64 | -0.26 | -14.08 | 13.55 | 0.89 | 4.81±9.23 | 1.72±0.66 | 1.39±0.15 | 0.31** | -0.93 | 1.55 | 0.01 | 21.79±15.29 |
| - *standing* | 69.69±17.02 | 66.69±13.84 | 3* | -12.34 | 18.34 | 0.85 | 7.36±6.73 | 1.51±0.43 | 1.47±0.27 | 0.08 | -0.67 | 0.83 | 0.42 | 18.03±13.68 |
| *Low intensity household activities* |  |  |  |  |  |  |  |  |  |  |  |  |  |  |
| - *Cooking* | 74.02±17.20 | 72.27±13.42 | 1.75 | -24.43 | 27.93 | 0.62 | 11.22±12.84 | 2.36±0.96 | 2.29±0.89 | 0.04 | -1.95 | 2.03 | 0.40 | 34.20±37.71 |
| - *Cleaning the table* | 73.97±17.84 | 77.53±13.58 | -3.56 | -27.66 | 20.54 | 0.67 | 14.17±14.38 | 3.56±1.26 | 2.94±1.18 | 0.51** | -1.65 | 2.67 | 0.51 | 32.87±26.08 |
| *Moderate intensity household activities* |  |  |  |  |  |  |  |  |  |  |  |  |  |  |
| - *Vacuum cleaning* | 79.44±17.77 | 78.63±14.23 | 0.81 | -24.46 | 26.08 | 0.68 | 12.25±11.04 | 3.82±1.33 | 3.12±1.22 | 0.62** | -1.97 | 3.21 | 0.35 | 30.09±22.00 |
| - *Climbing the stairs* | 88.35±19.92 | 85.27±21.93 | 6.71 | -32.69 | 38.84 | 0.63 | 19.15±11.56 | 4.26±1.70 | 2.97±1.23 | 1.22* | -2.64 | 5.09 | 0.03 | 40.00±24.35 |
| - *Walking down the stairs* | 88.95±19.22 | 88.90±17.60 | 0.048 | -23.42 | 23.52 | 0.81 | 8.66±9.17 | 5.15±1.35 | 3.81±1.18 | 1.32** | -1.37 | 4.01 | 0.12 | 27.63±18.04 |
| *Cycling* |  |  |  |  |  |  |  |  |  |  |  |  |  |  |
| - *cycling at 0 w (70 rpm)* | 73.81±16.94 | 74.59±13.81 | -0.78 | -23.35 | 21.79 | 0.73 | 11.19±14.37 | 2.84±0.77 | 2.62±1.21 | 0.18 | -1.92 | 2.29 | 0.43 | 27.81±26.50 |
| - *cycling at 50 w (70 rpm)* | 82.24±16.52 | 76.38±17.91 | 4.85* | -20.33 | 30.04 | 0.67 | 13.47±12.22 | 3.76±1.27 | 3.05±1.64 | 0.71* | -2.49 | 3.91 | 0.28 | 36.55±32.93 |
| - *cycling at 100 w (70 rpm)* | 92.32±20.68 | 81.50±21.17 | 10.82** | -22.33 | 43.98 | 0.51 | 18.02±14.07 | 5.38±2.18 | 3.97±2.29 | 1.37** | -3.66 | 6.41 | 0.20 | 44.18±39.62 |
| *Walking* |  |  |  |  |  |  |  |  |  |  |  |  |  |  |
| - *walking at 4 km/h and 5%* | 89.84±19.81 | 80.99±19.58 | 8.85** | -29.99 | 47.69 | 0.39 | 19.57±15.92 | 5.14±2.13 | 2.96±1.77 | 2.03* | -3.34 | 7.40 | 0.12 | 54.43±38.72 |
| - *walking at 5.5 km/h and 5% slope* | 95.15±21.34 | 83.91±21.82 | 11.24** | -29.77 | 52.24 | 0.39 | 19.82±16.28 | 5.92±2.22 | 3.19±1.89 | 2.61** | -2.70 | 7.91 | 0.12 | 53.73±37.26 |
| - *walking at 4 km/h and 10% slope* | 95.90±21.40 | 87.02±23.55 | 8.88* | -36.42 | 54.17 | 0.40 | 19.94±17.15 | 6.13±2.61 | 3.87±2.42 | 2.18** | -4.67 | 9.02 | 0.10 | 59.00±55.93 |
| *Total protocol*  *(resting time included)* | 80.97±20.78 | 80.19±19.54 | 0.78 | -34.46 | 36.01 | 0.60 | 16.20±17.21 | 3.80±2.11 | 2.96±1.71 | 0.79** | -3.53 | 5.10 | 0.26 | 42.87±38.51 |

*Table S3. Accuracy of HR and EE measurements by PHB for recreational athletes*

Mean differences are calculated as device minus criterion measure.

SD: Standard Deviation

LoA: Limits of Agreement

ICC: Intraclass correlation coefficient

MAPE: Mean Average Percentage Error

OM: Oxycon Mobile

PHB: Philips Health Band

BPM: Beats Per Minute

Kcal: Kilocalories

*p<0.05, **p<0.001

| Group and activity | OM, Mean difference HR [BPM] | P-value | PHB, Mean difference HR [BPM] | P-value | OM, Mean difference EE [kcal] | P-value | PHB Mean difference EE [kcal] | P-value |
| --- | --- | --- | --- | --- | --- | --- | --- | --- |
| ***CAD Cycling*** |  |  |  |  |  |  |  |  |
| - *0 vs 40 watts* | -7.13 | <0.001 | -0.55 | 0.63 | -0.51 | <0.001 | -0.17 | 0.38 |
| - *0 vs 70 watts* | -13.37 | <0.001 | -4.32 | 0.003 | -1.34 | <0.001 | -0.74 | <0.001 |
| - *40 vs 70 watts* | -6.24 | <0.001 | -3.76 | <0.001 | -0.93 | <0.001 | -0.57 | <0.001 |
| ***CAD Walking*** |  |  |  |  |  |  |  |  |
| - *4 km/h vs 5.5 km/h* | -5.17 | <0.001 | -2.20 | 0.051 | -0.58 | <0.001 | -0.02 | 0.89 |
| - *4 km/h vs 4km/h 5% slope* | -4.28 | <0.001 | -4.26 | <0.001 | -0.58 | 0.003 | -0.19 | 0.19 |
| - *5.5 km/h vs 4 km/h 5% slope* | 1.49 | 0.05 | -2.07 | 0.074 | 0.01 | 0.51 | -0.16 | 0.30 |
| ***HFrEF Cycling*** |  |  |  |  |  |  |  |  |
| - *0 vs 25 watts* | 0 | 1 | 0.46 | 0.57 | -0.16 | 0.32 | -0.02 | 0.79 |
| - *0 vs 50 watts* | -4.32 | 0.12 | -0.82 | 0.35 | -0.93 | <0.001 | -0.44 | <0.001 |
| - *25 vs 50 watts* | -4.32 | 0.01 | -1.28 | 0.02 | -0.84 | <0.001 | -0.41 | <0.001 |
| ***HFrEF Walking*** |  |  |  |  |  |  |  |  |
| - *2 km/h vs 4 km/h* | -4.68 | <0.001 | -3.78 | 0.009 | -0.77 | <0.001 | -0.13 | 0.50 |
| - *2 km/h vs 2 km/h 5% slope* | -1.99 | <0.001 | -2.60 | 0.015 | -0.20 | 0.086 | 0.11 | 0.54 |
| - *4 km/h vs 2 km/h 5% slope* | 2.72 | <0.001 | 1.18 | 0.37 | 0.54 | <0.001 | 0.24 | 0.11 |
| ***Recretional Athletes Cycling*** |  |  |  |  |  |  |  |  |
| - *0 vs 50 watts* | -7.43 | <0.001 | -2.06 | 0.14 | -0.91 | <0.001 | -0.36 | 0.003 |
| - *0 vs 100 watts* | -18.51 | <0.001 | -7.07 | <0.001 | -2.54 | <0.001 | -1.31 | <0.001 |
| - *50 vs 100 watts* | -11.09 | <0.001 | -5.01 | <0.001 | -1.70 | <0.001 | -0.95 | <0.001 |
| ***Recreational Athletes Walking*** |  |  |  |  |  |  |  |  |
| - *4 km/h 5% slope vs 5.5 km/h 5% slope* | -5.31 | <0.001 | -3.51 | 0.005 | -0.80 | <0.001 | -0.24 | 0.08 |
| - *4 km/h 5% slope vs 4 km/h 10% slope* | -6.06 | <0.001 | -6.35 | <0.001 | -1.05 | <0.001 | -0.76 | <0.001 |
| - *5.5 km/h 5% slope vs 4 km/h 10% slope* | -0.75 | 0.41 | -2.84 | 0.02 | -0.26 | 0.14 | -0.52 | 0.01 |

*Table S4. Responsiveness of OM and PHB*

CAD: Coronary Artery Disease

HFrEF: Heart Failure with Reduced Ejection Fraction

OM: Oxycon Mobile

PHB: Philips Health Band

BPM: Beats Per Minute

Kcal: Kilocalories
